# Efficacy of Platelet Rich Plasma Therapy in Melasma Using Microinjections and Microneedling Techniques

**DOI:** 10.1111/jocd.70246

**Published:** 2025-05-21

**Authors:** Seema Sitaula, Shree Ram Pokhrel, Prayash Paudel, Aabha Shrestha

**Affiliations:** ^1^ Department of Dermatology Tribhuvan University Teaching Hospital Kathmandu Nepal; ^2^ Bachelor of Medicine Bachelor of Surgery, Maharajgunj Medical Campus Tribhuvan University Teaching Hospital Kathmandu Nepal; ^3^ Doctor of Medicine in Department of Dermatology and Venerology Tribhuvan University Teaching Hospital Kathmandu Nepal

**Keywords:** melasma, microinjection, microneedling, PRP therapy

## Abstract

**Introduction:**

Melasma is a long‐term acquired disorder characterized by symmetrical darkening in facial regions exposed to sunlight. Although some risk factors have been identified, the etiology remains unclear, and interventions frequently do not totally improve outcomes.

**Aim:**

It compares the efficacy and side effects of platelet‐rich plasma therapy administered by microneedling versus microinjection to detect any difference in the delivery methods, treatment outcomes, and safety profiles.

**Methods:**

In this prospective, single‐center, randomized split‐face research, 62 patients' melasma was treated with PRP therapy using microneedling versus microinjection. Microneedling and microinjection were used to apply PRP to the face's two sides. Photographs were obtained before therapy, and the modified Melasma Area and Severity Index (MASI), Physician Global Assessment, and Patient Global Assessment were used for monthly follow‐up evaluation. In order to compare safety and efficacy, adverse events were recorded and statistical analysis was performed.

**Results:**

A significantly significant difference (*p* < 0.001) was observed in the mean change in MASI score between microinjection and microneedling. The microneedling group showed a 73.33% improvement while the microinjection group had an 18.33% improvement, both of which were greater than 50%. The microneedling group showed a 46.67% improvement and the microinjection group had a 5% improvement of over 75%. There were no noteworthy negative effects observed.

**Conclusion:**

PRP therapy with microneedling is superior to microinjection in treating melasma; patients who receive microneedling show noticeably better improvement.

## 1 Introduction

A prevalent dermatological issue among Asians, melasma is an acquired hyperpigmentary skin condition that usually manifests as symmetrical hyperpigmented macules and patches on the forehead, nose, and bilateral cheeks.

Although men might also have the illness, women are primarily impacted [1, 2]. Although hormonal imbalances and UV exposure have been recognized as the main contributing causes to the complex etiopathogenesis of melasma, cutaneous melanin deposition brought on by disturbance of the basement membrane has recently been proposed as a potential contributing component [2, 3].

Treating melasma poses a significant challenge due to its resistance to therapies and frequent relapses or recurrences. Oral drugs, topical bleaching agents, chemical peels, and laser or light‐based therapies are some of the current treatments available; however, each has drawbacks, different success rates, and possible side effects [4]. In this context, platelet‐rich plasma (PRP) therapy has become a viable treatment option for melasma, either alone or in combination.

PRP is a condensed form of autologous plasma obtained through centrifugation, enriched with a high number of platelets. It has broad medical and aesthetic applications in dermatology [5]. Growth factors such as TGF‐β1 and PDGF in PRP have been found to reduce melasma by decreasing melanogenesis through delayed extracellular signal‐regulated kinase activation [6]. PRP is increasingly recognized as a novel intervention with potential benefits for both the treatment and long‐term management of melasma.

However, the deeper and more reliable delivery of medication made possible by the microchannels formed during the microneedling procedure may be the reason for the better therapeutic response seen in the microneedling group when comparing the two delivery methods (microinjection and microneedling) [7].

## 2 Materials and Methods

This study examined the safety and effectiveness of administering PRP therapy for the treatment of melasma using microneedling and microinjection procedures. It was a single‐center, prospective, randomized trial of a split‐face, comparative study. This study is approved by the Institutional Review Committee(IOM) and all procedures followed ethical guidelines. Written informed consent was obtained from all 62 participants before their enrollment in the study. Each patient underwent a total of three treatment sessions, scheduled once a month over a period of 3 months. Patients did not use any topical treatment between sessions.

### 2.1 Pre‐Treatment Assessment

Every participant had a thorough pre‐treatment assessment. To ascertain the histological type of melasma (epidermal, dermal, or mixed), a thorough medical history, a general examination, and a dermatological evaluation using Wood's Lamp were all part of this process. As part of the pre‐treatment assessment, a full blood count (FBC), including platelet count, was checked to gather baseline health data and ensure safe participation in the study. In order to accurately compare the results after therapy, pre‐treatment photos taken from various angles were used to document the degree of melasma.

### 2.2 Treatment Protocol

Two groups were formed from the participants. PRP was applied topically after patients had microneedled with a 1.5 mm needle depth on the left side of their faces. A microneedling pen equipped with a cartridge containing 24 needles was used for this procedure. PRP microinjections were applied to the right side of the face using a 1.5 mm needle depth.

Prior to PRP administration, 0.1 mL of intradermal PRP was injected as part of a reactivity test. After 15 min, the skin was checked for signs of redness or whealing. If there were no negative reactions, the process continued.

### 2.3 Anesthesia and PRP Preparation

Twenty minutes prior to the treatment, a topical anesthetic cream containing 5% lidocaine was administered to the patient's face to ensure their comfort. Each participant had 10 mL of autologous whole blood extracted and processed for PRP production. To separate the plasma, the blood was centrifuged for 10 min at 1500 rpm. The platelets were then concentrated to a level 4.5 times higher than baseline (8–9 lakhs/μL) by centrifuging the blood again for 10 min at 3700 rpm. Prior to application, the platelets were resuspended using platelet‐poor plasma.

### 2.4 Outcome Measures

To evaluate the results of treatment, both subjective and objective assessments were used.

By assessing the extent, intensity, and distribution of pigmentation, the modified Melasma extent and Severity Index (MASI) was used to gauge the severity of melasma. For visual comparison, pictures were taken before and after the treatment. Additionally, participants were requested to record any discomfort, negative effects, or advancements. The treatment response was categorized as follows at the end of the study: no response (no improvement), mild response (less than 25% improvement), moderate response (25% to less than 50% improvement), good response (50% to less than 75% improvement), and very good response (more than 75% improvement).

### 2.5 Follow‐Up and Data Analysis

Patients were monitored for possible adverse effects, any recurrence of melasma, and the outcomes at regular intervals. The effectiveness and safety of PRP treatments administered by microneedling and microinjection were compared using statistical analysis of the data. Using the proper tests, statistical significance was established; a *p*‐value of less than 0.05 was deemed significant.

## 3 Results

Out of the 60 patients included in the study, most (63.33%) belonged to the age group of 30–50 years. There was a notable gender disparity, with significantly more females (53) compared to males (7) [Table 1].

**TABLE 1:** Age and sex distribution.

Age grouped	Female count	Female percentage	Male count	Male percentage	Total
10–20	4	6.67	0	0	4
20–30	10	16.67	2	3.33	12
30–40	20	33.33	2	3.33	22
40–50	14	23.33	2	3.33	16
50–60	3	5	1	1.67	4
60–70	2	3.33	0	0	2
Total	53	88.33	7	11.67	60

The majority of patients had Fitzpatrick skin types 4 or 5, while only one patient had skin type 1 [Tables 2 and 3].

**TABLE 2:** Fitzpatrick skin type and patterns of melasma.

Fitzpatrick skin type	Count	Percentage
3	1	1.67
4	45	75
5	14	23.33
Total	60	100

**TABLE 3:** Patterns of melasma.

Patterns of melasma	Count	Percentage
Dermal	17	28.33
Epidermal	15	25
Mixed	28	46.67
Total	60	100

Regarding the type of melasma, 46.67% of patients were diagnosed with the mixed type, 25% had the epidermal type, and 28.33% had the dermal type. Additionally, 63.3% of patients reported a family history of melasma, highlighting a possible genetic predisposition [Table 4].

**TABLE 4:** Clinical data.

Parameter	*n* (%)
*Family history*	
Positive	38 (63.3)
Negative	22 (36.7)
*History of oral contraceptive use*	
Positive	8 (13.3)
Negative	52 (86.7)
*History of thyroid disease*	
Positive	8 (13.3)
Negative	52 (86.7)

The microinjection group's overall MASI scores decreased from 229.9 at the initial visit to 131.2 at the final follow‐up, indicating improved treatment outcomes. A statistically significant improvement of 36.6% was shown by the mean MASI score, which decreased from 5.013 to 2.187 (*p* < 0.01). The microneedling group, on the other hand, showed a larger decline in overall MASI scores, going from 229.9 at baseline to 72.9 at the last follow‐up. A 70.21% improvement was observed in the mean MASI score, which decreased from 3.832 to 1.215 (*p* < 0.001) [Table 5].

**TABLE 5:** Mean MASI scores, percentage improvement, and *p*‐value of both groups.

	Before	After
*Microinjection*		
Total MASI	300.8	131.2
Mean	5.013 ± 0.12996	2.187 ± 1.7886
Percentage improvement	36.6%	
*p*‐value	< 0.001	
*Microneedling*		
Total MASI	229.9	72.9
Mean	3.832 ± 1.3681	1.215 ± 1.0925
Percentage improvement	70.21%	
*p*‐value	< 0.001	

Microneedling was substantially more successful than microinjection when the two groups were compared (*p* < 0.001). More than 50% improvement was attained by 18.33% of patients in the microinjection group and 73.33% of patients in the micro needling group. Additionally, compared to just 5% in the microinjection group, 46.67% of patients in the microneedling group demonstrated more than 75% improvement [Table 6].

**TABLE 6:** Percentage improvement of MASI scores in both the groups.

Response percentage	Count	Microinjection	Count	Microneedling
< 25	25	45.5%	1	1.7%
25–50	19	34.5%	15	25%
50–75	8	14.5%	16	26.7%
> 75	3	5.5%	28	46.7%

**FIGURE 1:**
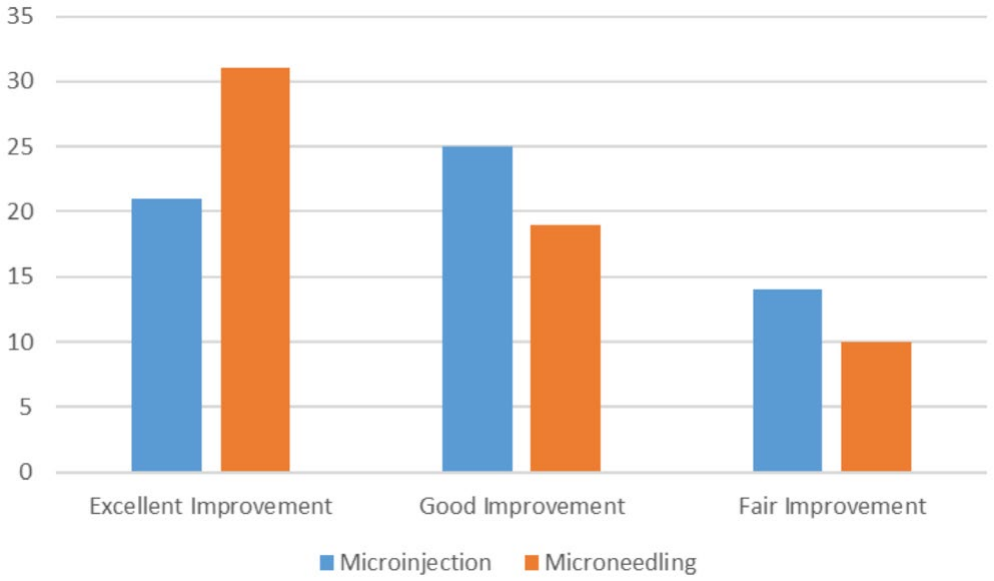
PGA after microinjection and microneedling.

**FIGURE 2:**
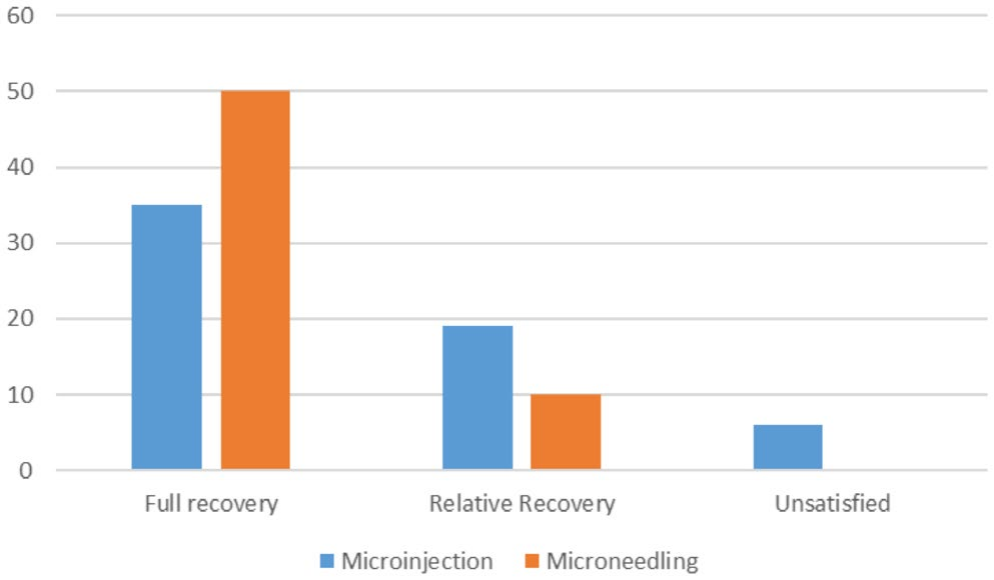
pTGA after microinjection and microneedling.

Clinical photos and patient satisfaction ratings (PtGA and PGA) verified that both groups had significantly improved. Crucially, the treatments were well tolerated; most patients experienced very minor side effects, such as soreness, burning, and redness, which went away in 1–2 days. Total PtGA and total PGA scores are shown in [Figures 1 and 2].

## 4 Discussion

The effectiveness of platelet‐rich plasma therapy delivered by microneedling as opposed to microinjections in the management of melasma is evaluated in this study. In 2020, Punyaphat et al. carried out a pilot research in Thailand [2], the first randomized placebo‐controlled trial, which concluded that intradermal PRP injection can serve as a melasma adjuvant or alternative treatment. As far as we are aware, this is the first study conducted in Nepal analyzing the efficacy of PRP in melasma and also the first one to compare different modalities of PRP therapy in patients. Among 60 patients included in our study, 11.67% were male, 75% of patients had Fitzpatrick skin type 4, and most of them, 46.67%, had a mixed pattern of melasma.

Improvement was evaluated by PtGA, PGA, MASI, and clinical photos. Both the first and last visits were used to evaluate MASI. PGA was measured by evaluating a patient's condition based on clinical observation and standard criteria. PTGA is a subjective measure where patients evaluate their own condition and improvement based on their experiences with treatment. There was a reduction in MASI, and improvement of PGA and PtGA over time. Our results indicate that while both modes of delivery result in improvement, PRP with microneedling yields better results.

Microneedling significantly outperformed microinjection in improving MASI scores in patients with melasma. A remarkable 46.7% of participants in the microneedling group achieved > 75% improvement, compared to only 5.5% in the microinjection group. However, a comparable study conducted in India revealed that at the conclusion of the third follow‐up visit, the MASI score improved 35.72% in the microinjection group and 44.41% in the microneedling group. Twelve patients (41.38%) in the microneedling group and six patients (26.09%) in the microinjection group demonstrated improvements of greater than 50%. When comparing the mean difference between the microinjection and microneedling groups, the improvement was statistically significant (*p* < 0.001) [7].

The possible reason could be the more uniform and deeper penetration of molecules due to the microchannels provided during microneedling, as evidenced by similar results in studies using microneedling for drug delivery [7]. Lima et al. (2017) [8] reported that microneedling promotes dermal remodeling and enhances the delivery of depigmenting agents, making it particularly effective in treating pigmentation disorders, including melasma. Bailey et al. [9] also reported similar findings. He came to the conclusion that topical therapy combined with microneedling improved the severity of melasma more than topical therapy alone, with a modest effect at 8 weeks and a big effect at 12–16 weeks. Microneedling in combination with triple combination therapy was also proven by Emerson et al. [8] in his study.

Conversely, microinjections, which involve the localized delivery of agents like tranexamic acid, are moderately effective in mild‐to‐moderate cases but demonstrate limited efficacy in severe melasma by Lee et al., 2006 [10]. These results affirm that microneedling offers a superior therapeutic option for melasma management, particularly in achieving significant improvements in MASI scores. The improvement between the microinjection and microneedling groups was statistically significant (*p* < 0.001).

The results of this study's Physician's Global Assessment (PGA) and Patient's Global Assessment (pTGA) show that microneedling is clearly superior to microinjection for the treatment of melasma. These findings demonstrate that microneedling is more effective than microinjection from the viewpoints of the doctor (PGA) and the patient (pTGA), demonstrating its capacity to produce notable enhancement and increased patient satisfaction. The consistent results between PGA and pTGA support microneedling as a more effective melasma treatment strategy [7].

PRP's abundant concentration of growth factors, including platelet‐derived growth factor, transforming growth factor‐beta, and vascular endothelial growth factor, contributes to its multifactorial mechanism of action in the treatment of melasma. These growth factors have crucial roles in tissue regeneration, collagen synthesis, and angiogenesis and may contribute to the improvement of melasma by promoting epidermal turnover, modulating melanocyte activity, and influencing inflammatory responses in the skin. PRP has been found to be efficacious in multiple studies; one such study is by Sirithanabadeekul et al. [2] in the year 2020. The microneedling process itself further enhances the therapeutic effect by stimulating collagen production and tissue remodeling. Various studies have been done on PRP applications in dermatological conditions. For instance, when treating melasma, Hofny et al. [11] contrasted the administration of PRP via microneedling and microinjections. These studies are important but do not directly address the issue of the comparative efficacy of PRP with microneedling versus microinjections in melasma, which is what our research is based on. Gharib et al. [12] concluded that PRP with microneedling is more efficacious than microneedling with Tranexamic acid.

Our study's open‐label approach and small sample size are among its limitations; these could have created biases and limited generalizability. Larger studies, randomized and controlled, must be conducted in order for these results to be validated. Further research parameters regarding optimal PRP delivery in treating melasma would include PRP concentration, injection depth, and treatment frequency.

Regardless of these limitations, our study adds to the expanding literature that supports the role of PRP in managing melasma. The relative noninvasiveness of both the microneedling and microinjection techniques, in addition to the possible ability for significant clinical improvement, makes PRP therapy quite attractive for patients considering its potential for melasma. A future line of inquiry exploring various combinations and treatments based on the characteristics of the individual melasma will be of essential value to optimally treat this multifactorial dermatosis with a sophisticated pathophysiology.

## 5 Melasma Before and After

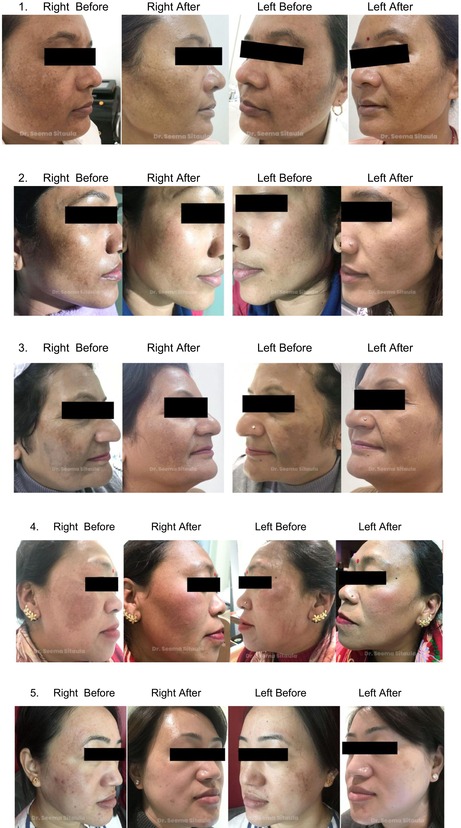

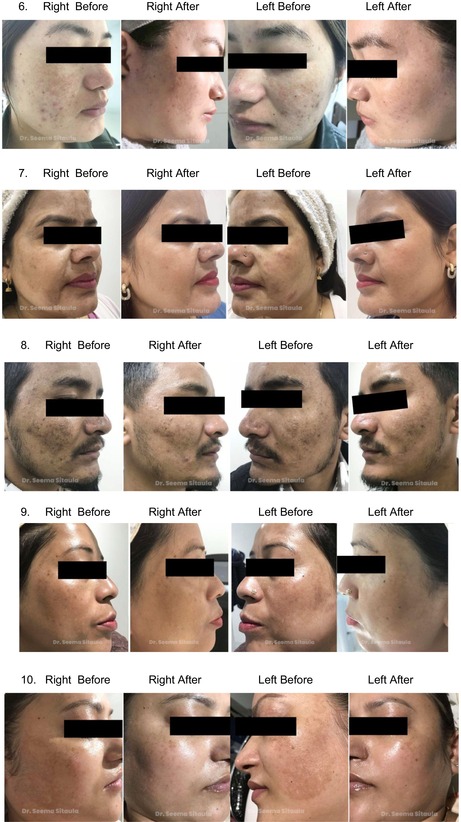

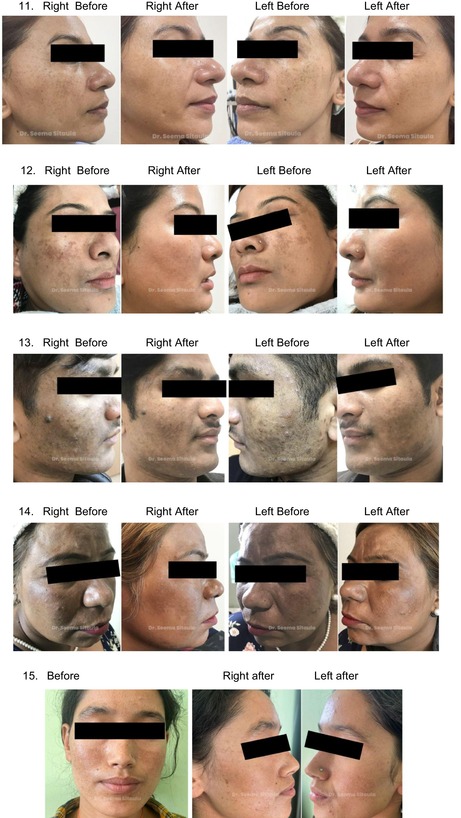

## Conflicts of Interest

The authors declare no conflicts of interest.

## Data Availability

The data that support the findings of this study are available from the corresponding author upon reasonable request.

## References

[1] S. Rajanala , M. B. d. C. Maymone , and N. A. Vashi , “Melasma Pathogenesis: A Review of the Latest Research, Pathological Findings, and Investigational Therapies,” Dermatology Online Journal 25, no. 10 (2019): 1. 31735001

[2] P. Sirithanabadeekul , A. Dannarongchai , and A. Suwanchinda , “Platelet‐Rich Plasma Treatment for Melasma: A Pilot Study,” Journal of Cosmetic Dermatology 19, no. 6 (2020): 1321–1327. 31568636 10.1111/jocd.13157

[3] S. H. Kwon , J. I. Na , J. Y. Choi , and K. C. Park , “Melasma: Updates and Perspectives,” Experimental Dermatology 28, no. 6 (2019): 704–708. 30422338 10.1111/exd.13844

[4] I. Arellano , T. Cestari , J. Ocampo‐Candiani , et al., “Preventing Melasma Recurrence: Prescribing a Maintenance Regimen With an Effective Triple Combination Cream Based on Long‐Standing Clinical Severity,” Journal of the European Academy of Dermatology and Venereology 26, no. 5 (2012): 611–618. 21623930 10.1111/j.1468-3083.2011.04135.x

[5] L. Zhao , M. Hu , Q. Xiao , et al., “Efficacy and Safety of Platelet‐Rich Plasma in Melasma: A Systematic Review and Meta‐Analysis,” Dermatology and Therapy 11, no. 5 (2021): 1587–1597. 34269967 10.1007/s13555-021-00575-z PMC8484406

[6] E. R. M. Hofny , M. R. A. Hussein , A. Ghazally , A. M. Ahmed , and A. A. Abdel‐Motaleb , “Increased Expression of TGF‐β Protein in the Lesional Skins of Melasma Patients Following Treatment With Platelet‐Rich Plasma,” Journal of Cosmetic and Laser Therapy 21, no. 7–8 (2019): 382–389. 31554441 10.1080/14764172.2019.1668016

[7] L. Budamakuntla , E. Loganathan , D. H. Suresh , et al., “A Randomised, Open‐Label, Comparative Study of Tranexamic Acid Microinjections and Tranexamic Acid With Microneedling in Patients With Melasma,” Journal of Cutaneous and Aesthetic Surgery 6, no. 3 (2013): 139–143. 24163529 10.4103/0974-2077.118403 PMC3800287

[8] E. V. A. Lima , M. M. D. A. Lima , M. P. Paixão , and H. A. Miot , “Assessment of the Effects of Skin Microneedling as Adjuvant Therapy for Facial Melasma: A Pilot Study,” BMC Dermatology 17, no. 1 (2017): 14. 29183309 10.1186/s12895-017-0066-5 PMC5706369

[9] A. J. M. Bailey , H. O. Y. Li , M. G. Tan , W. Cheng , and J. S. Dover , “Microneedling as an Adjuvant to Topical Therapies for Melasma: A Systematic Review and Meta‐Analysis,” Journal of the American Academy of Dermatology 86, no. 4 (2022): 797–810. 33857549 10.1016/j.jaad.2021.03.116

[10] J. H. Lee , J. G. Park , S. H. Lim , et al., “Localized Intradermal Microinjection of Tranexamic Acid for Treatment of Melasma in Asian Patients: A Preliminary Clinical Trial,” Dermatologic Surgery 32, no. 5 (2006): 626–631. 16706756 10.1111/j.1524-4725.2006.32133.x

[11] E. R. M. Hofny , A. A. Abdel‐Motaleb , A. Ghazally , A. M. Ahmed , and M. R. A. Hussein , “Platelet‐Rich Plasma Is a Useful Therapeutic Option in Melasma,” Journal of Dermatological Treatment 30, no. 4 (2019): 396–401. 30220226 10.1080/09546634.2018.1524821

[12] K. Gharib , F. F. Mostafa , and S. Ghonemy , “Therapeutic Effect of Microneedling With Platelet‐Rich Plasma Versus Microneedling With Tranexamic Acid for Melasma,” Journal of Clinical and Aesthetic Dermatology 14, no. 8 (2021): 44–48. PMC8570658 34840657

